# Enhanced immunoregulation of mesenchymal stem cells by IL-10-producing type 1 regulatory T cells in collagen-induced arthritis

**DOI:** 10.1038/srep26851

**Published:** 2016-06-01

**Authors:** Jung-Yeon Lim, Keon-Il Im, Eun-Sol Lee, Nayoun Kim, Young-Sun Nam, Young-Woo Jeon, Seok-Goo Cho

**Affiliations:** 1 Institute for Translational Research and Molecular Imaging, Seoul St. Mary’s Hospital, The Catholic University of Korea College of Medicine, Seoul, 137-701, Republic of Korea; 2 Laboratory of Immune Regulation, Convergent Research Consortium for Immunologic Disease Seoul St. Mary’s Hospital, The Catholic University of Korea College of Medicine, Seoul, 137-701, Republic of Korea; 3 Department of Hematology, Catholic Blood and Marrow Transplantation Center, Seoul St. Mary’s Hospital, The Catholic University of Korea College of Medicine, Seoul, 137-701, Republic of Korea

## Abstract

Mesenchymal stem cells (MSCs) possess immunomodulatory properties and have potential, however, there have been conflicting reports regarding their effects in rheumatoid arthritis (RA), which causes inflammation and destruction of the joints. Through a comparative analysis of regulatory T (Treg) and IL-10-producing type 1 regulatory T (Tr1) cells, we hypothesized that Tr1 cells enhance the immunoregulatory functions of MSCs, and that a combinatorial approach to cell therapy may exert synergistic immunomodulatory effects in an experimental animal model of rheumatoid arthritis (RA). A combination of MSCs and Tr1 cells prevented the development of destructive arthritis compared to single cell therapy. These therapeutic effects were associated with an increase in type II collagen (CII)-specific CD4+CD25+Foxp3+ Treg cells and inhibition of CII-specific CD4+IL-17+ T cells. We observed that Tr1 cells produce high levels of IL-10-dependent interferon (IFN)-β, which induces toll-like receptor (TLR) 3 expression in MSCs. Moreover, induction of indoleamine 2,3-dioxygenase (IDO) by TLR3 involved an autocrine IFN-β that was dependent on STAT1 signaling. Furthermore, we observed that production of IFN-β and IL-10 in Tr1 cells synergistically induces IDO in MSCs through the STAT1 pathway. These findings suggest co-administration of MSCs and Tr1 cells to be a novel therapeutic modality for clinical autoimmune diseases.

Rheumatoid arthritis (RA) is an autoimmune disease that causes chronic inflammation of the joints involving local production of pro-inflammatory cytokines, such as interleukin (IL)-1, tumor necrosis factor-alpha (TNF-α), IL-6, and IL-17^1^,^2^. In particular, T helper (Th) 17 cells are involved in the induction and progression of various pathologies, whereas Foxp3+ regulatory T (Treg) cells inhibit autoimmunity and are responsible for tolerance against self-antigens^3^. During the progression of this disease, continuous inflammatory responses take place at the synovial membrane, contributing to joint destruction/deformation and cartilage damage due to the pathologic proliferation of synoviocytes^1^. Therefore, RA therapy aims to suppress the production of pro-inflammatory cytokines and joint destruction and, thus, prevent long-term disability. Several general classes of drugs are commonly used in the treatment of RA, including nonsteroidal anti-inflammatory drugs (NSAIDs)^4^,^5^, corticosteroids^6^, and disease-modifying anti-rheumatic drugs (DMARDs)^7^. Although various RA medications can limit the progressive articular damage caused by inflammatory cells and synoviocytes, moderate or severe side effects, including diarrhea, skin rash and an increased susceptibility to infections, are observed at higher doses or following long-term use^8^. Therefore, novel approaches to treating this disease are required.

In the preclinical and/or the clinical setting, bone marrow (BM)-MSCs have shown promising results in research and in clinical trials, including those related to autoimmune diseases, graft-versus-host disease following bone marrow transplantation, cardiovascular diseases, orthopedic injuries, cardiovascular diseases, organ transplantation, and liver diseases^9^,^10^,^11^,^12^. Immunoregulation by MSCs is mediated directly by cell–cell contact or indirectly by secretion of immunomodulatory factors, such as prostaglandin E_2_ (PGE_2_), indoleamine 2,3-dioxygenase (IDO), and transforming growth factor-beta (TGF-β)^13^. In addition, previous studies have indicated that toll-like receptor (TLR) activation induces the production of downstream cytokines in MSCs^14^. MSCs can be differently polarized by TLR ligands into two acting phenotypes—TLR4 agonists induce a pro-inflammatory MSC1 phenotype, while TLR3 configures MSCs towards the immunosuppressive MSC2 phenotype. According to this paradigm, MSC1s secrete high levels of IL-6, IL-8 or TGF-β, while MSC2s produce increased levels of IL-10, IDO and PGE_2_^14^. Therefore, the therapeutic potential of MSCs *in vivo* can be modulated by exposing them to TLR ligands^13^.

The therapeutic potential of MSCs in preclinical studies is controversial, which may have delayed their evaluation in clinical trials. While some studies have demonstrated the efficacy of MSC therapy in an experimental model of RA^15^, other groups have suggested that MSCs alone do not suppress the development of Th17 and TNF-α-mediated joint inflammation^16^,^17^. We have also observed that MSCs are ineffective in a murine model of CIA^18^. Therefore, a better understanding of the immunological effects of MSCs by environmental stimuli will facilitate development of efficacious MSC-based cell therapies.

Several subsets of regulatory T cells with distinct phenotypes and mechanisms of action have been identified. These cells include CD4+CD25+Foxp3+ regulatory T (Treg) and/or IL-10-producing type 1 regulatory T (Tr1) cells and have been shown to play an important role in T cell homeostasis and maintenance of immune responses, including the prevention of autoimmunity and inflammation^19^,^20^,^21^,^22^. *In vitro* and *in vivo* studies suggest that MSCs can generate Treg cells; indeed, the immunosuppressive effects of MSCs may depend on their effects on Treg generation or function^23^. Thus, a conditional microenvironment containing subsets of regulatory T cells plays an important role in the function and behavior of MSCs.

Based on these reports, the aim of the present study was to perform a comparative analysis of MSCs plus Treg and MSCs plus Tr1 cells *in vitro*. This is, to our knowledge, the first demonstration of the enhancement of the immunoregulatory functions of MSCs by Tr1 cells in a murine model of autoimmune arthritis; our findings identify the mechanism by which MSCs and Tr1 cells control the overwhelming inflammatory process. Thus, co-administration of MSCs and Tr1 cells may result in greater therapeutic efficacy against RA.

## Results

### Phenotypes of culture-expanded MSCs

MSCs were obtained from DBA1J mice bone marrow showing typical fibroblast-like morphology, as described previously^18^. Flow cytometric analysis demonstrated that MSCs did not express c-kit, CD11b, CD34, CD106, CD45, CD31, CD80, or CD86, but expressed MSC-associated antigens, such as Sca-1, CD44 and CD29 (Supplementary Fig. 1A). Based on these results, we conclude that MSCs have the capacity to differentiate into osteocytes, chondrocytes and adipocytes *in vitro* (Supplemental Fig. 1B).

### Comparative analysis of culture-expanded Treg and Tr1 cells characteristics

**Figure 1:**
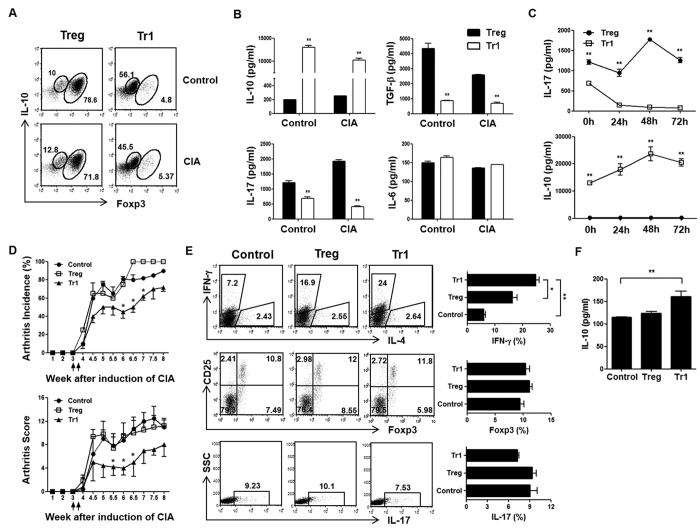
Characterization of culture-expanded Treg and Tr1 cells. Treg and Tr1 cells culture conditions: Treg CD4+Foxp3^high^IL-10^low^ cells (anti-IFN-γ, anti-IL-4, TGF-β, retinoic acid, anti-CD3, anti-CD28); Tr1 CD4+Foxp3^low^IL-10^high^ cells (Dex, Vit D3, anti-CD3, anti-CD28). (**A**) *In vitro* differentiation of Treg and Tr1 cells from splenic CD4+ T cells of control or CIA mice was analyzed by flow cytometry using intracellular Foxp3 and IL-10 in gated CD4+ T cell populations. (**B**) Levels of secreted IL-10, TGF-β, IL-17, and IL-6 (n = 5) in the *ex vivo* expanded Treg and Tr1 cells (differentiated with anti-IFN-γ, anti-IL-4, TGF-β, retinoic acid, anti-CD3, anti-CD28 and Dex, Vit D3, anti-CD3, anti-CD28, respectively) culture media were measured by ELISA. (**C**) Supernatant levels (n = 5) of IL-10 and IL-17 in the absence of stimulant at 0, 24, 48, and 72 h following differentiation for two days were measured by ELISA. (**D**) Mice were injected intravenously with 5 × 10^5^ Treg or Tr1 cells three weeks following primary immunization. Control mice received intravenous injections of an equal volume of PBS at the same time points. Clinical incidence (upper) and arthritis score (lower) in treated mice. Data represent the means ± standard error of the mean (SEM) of the arthritis scores of six mice per group. Incidence of arthritis with a score >4. Data are representative of two independent experiments (n = 6 per group) with similar results. (**E**) Four weeks after primary immunization, intracellular flow cytometry was performed to analyze IFN-γ, IL-17 and Foxp3 expression in the gated CD4+ T cell populations from CIA mice. Values represent the percentages of positive cells. (**F**) On day 7 following injection of PBS, Treg, or Tr1 cells in CIA mice, serum IL-10 were measured by ELISA. All graphs display the means ± SEM; results are representative of two independent experiments and statistical significance was determined by Student’s two-tailed *t*-test and ANOVA with Bonferroni correction for multiple comparisons (*p < 0.05; **p < 0.01).

We and others previously reported that Treg cells can be generated in the presence of anti-IFN-γ, anti-IL-4, TGF-β and retinoic acid^24^. Approximately 70–80% of cells were CD4+Foxp3+ T cells, and low production of CD4+IL-10+ T cells from control and CIA mice on a DBA1J background was reported. On the other hand, the development of Tr1 cells was induced by Dex and Vit D3, and secretion of high IL-10 levels and minimal amounts of CD4+Foxp3+ T cells was observed (Fig. 1A). Tr1 cells showed decreased secretion of TGF-β and IL-17 and significantly increased IL-10 production compared to Treg cells. However, no significant difference in IL-6 levels was observed (Fig. 1B). To further explore this observation, we investigated the continuous production of IL-10 and IL-17 in re-cultured Treg and Tr1 cells supernatants by removing the stimulant at various time points. As a result, Tr1 cells maintained the production of IL-10 following removal of stimulated culture supernatants, whereas IL-17 production was barely detectable compared to Treg cells (Fig. 1C).

To perform a comparative analysis on the effects of Treg and Tr1 cells on disease activity and progression of CIA, DBA1J mice were immunized with CII and treated with Treg or Tr1 cells. The therapeutic effects of Tr1-treated CIA mice induced a reduction in the incidence (Fig. 1D, upper) and severity of clinical arthritis (Fig. 1D, lower) compared to Treg-treated CIA mice. Furthermore, Tr1-treated CIA mice produced increased levels of IFN-γ (control vs. Tr1, Treg vs. Tr1), but Foxp3 and IL-17 levels were similar in CII-stimulated T cells in spleen, as compared to Treg-treated CIA mice (Fig. 1E). Also, our results indicate that Tr1 cell-treated CIA mice showed increased serum IL-10 levels, compared to the control, by ELISA (Fig. 1F). These results suggest that the development of arthritis was regulated by IL-10 production in Tr1 cell-treated CIA mice.

As reported previously, IFN-γ-licensed MSCs exhibit upregulated IDO activity^25^. These results demonstrate a role for IFN-γ in MSC-mediated immune suppression *in vivo*^26^. Moreover, IFN-γ-induced IDO suppresses inflammation by suppressing Th17 cells, which are induced during the pathogenesis of CIA^27^. We hypothesized that IFN-γ production following Tr1 cell therapy is a potent inducer of IDO *in vivo*; the expression of IDO could be increased by stimulating MSCs with IFN-γ, which is associated with the regulation of Th17 and Treg cells^28^,^29^.

### Effects of MSCs and Tr1 cells co-culture on CII-specific CD4 T cell proliferative responses

**Figure 2:**
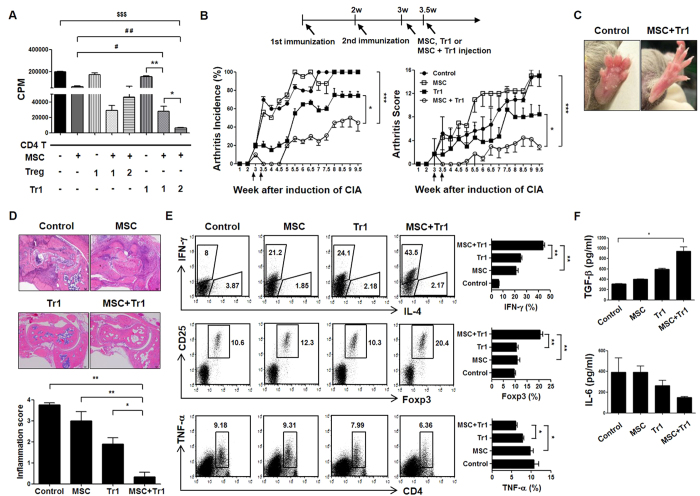
Suppression of CIA development by co-administration of MSCs and Tr1 cells. (**A**) Effector CD4+ T cell suppression in response to MSCs plus Treg cells or MSCs plus Tr1 cells was measured using ^3^H-thymidine incorporation in splenic cells isolated from CIA mice. 1: 1 × 10^4^; 2: 2 × 10^4^. (**B**) Mice (n = 6 per group) were injected with 5 × 10^5^ MSCs (i.p), 5 × 10^5^ Tr1 cells (i.v), or a co-infusion of MSCs and Tr1 cells twice weekly following secondary immunization. Clinical incidence (left) and arthritis scores (right) in treated mice. Data represent the means ± SEM of the arthritis scores of six mice per group. Incidence of arthritis with a score >4. Data are representative of two independent experiments with similar results. (**C**) Images from representative hind paws of CIA mice (left) and those treated with MSCs and Tr1 cells (right). Examples of hind limbs of CIA-affected mice. (**D**) Five weeks following the primary immunization, histological examination of joints obtained from each CIA mouse treated with MSCs in the absence of presence of Tr1 cells. Specimens were stained with H&E (×50). (**E**) Splenic cells were isolated from each group and analyzed using flow cytometry to examine IFN-γ, Foxp3 and TNF-α expression in the gated CD4+ T cell populations. (**F**) On day 7 following injection of PBS, MSCs, Tr1 cells, or a co-infusion of MSCs and Tr1 cells in CIA mice, levels of TGF-β (n = 4) and IL-6 (n = 4) in serum were measured using ELISA. All graphs display the means ± SEM; results are representative of two independent experiments and statistical significance was determined by Student’s two-tailed *t*-test and ANOVA with Bonferroni correction for multiple comparisons (*or ^#^*p* < 0.05; **or ^##^*p* < 0.01; ^$$$^*p* < 0.001).

As expected, very high ^3^H-thymidine incorporation was observed in the CII-specific T cell (1 × 10^5^) response, but co-culture of MSCs plus Treg or MSCs plus Tr1 cells resulted in a marked reduction in splenic T cell proliferation compared with single cells. Although there was no difference in CII-specific T cell proliferation between MSCs plus Treg (1 × 10^4^:1 × 10^4^ = 1:1) and MSCs plus Tr1 (1:1) cells, the suppressive effect was more prominent in splenic T cells from MSCs plus Tr1 cells ratio of 1:2 (1 × 10^4^:2 × 10^4^) compared to MSCs plus Tr1 cells ratio of 1:1 (1 × 10^4^:1 × 10^4^) following CII-specific stimulation (Fig. 2A). These data suggest that the combination of MSCs plus Tr1 cells more effectively regulates CII-specific stimulation.

### Therapeutic effect of MSCs and Tr1 cells on CIA development and severity

To explore the effects of co-infusion of MSCs and Tr1 cells on CIA disease activity and progression, DBA1J mice were immunized with CII and treated with MSCs in the absence or presence of Tr1 cells. Intraperitoneal (i.p.) injection of MSCs and intravenous (i.v.) injection of Tr1 cells reduced the clinical arthritis score (Tr1 vs. MSC+Tr1 (from 5.5 to 9.5 weeks), MSC vs. MSC+Tr1 (from 3.5 to 9.5 weeks)) and incidence of arthritis (Tr1 vs. MSC+Tr1 [from 7 to 9.5 weeks], MSC vs. MSC+Tr1 [from 4 to 9.5 weeks]) compared with single cell therapy (Fig. 2B). There was no significant difference between MSCs alone and Tr1 cells alone. Although a single infusion of Tr1 cells prevented the development of destructive arthritis in the early post-therapeutic period, these effects were generally mild and not prolonged. MSC therapy had no inhibitory effect at the early stage; these cells were in a worse condition (Fig. 2B). Co-infusion of MSCs plus Tr1 cells prevented the development of swelling or redness of the front or hind paws at five weeks following the first immunization (Fig. 2C). Regarding the histopathological features of the ankle joints, MSCs plus Tr1 cell-treated CIA mice showed a lower degree of inflammation, mononuclear cell infiltrate and pannus formation with superficial cartilage damage compared with controls and single cell therapy (Fig. 2D).

These data are in agreement with our hypothesis that the combination of MSCs plus Tr1 cells induces both CD4+IFN-γ+ and CD4+Foxp3+ T cells differentiation, and upregulation of CD4+TNF-α+ T cells are abolished following CII stimulation as compared to single cell therapy (Fig. 2E).

A dual role of TGF-β-induced anti-inflammatory Treg cells and pro-inflammatory Th17 cells from naïve T cells^30^ is indicated by the coordinate signaling by IL-6^31^. Serum levels of TGF-β and IL-6 were measured using ELISA to determine whether treatment with MSCs and Tr1 cells was associated with alterations in the cytokine response to T cells. Mice treated with MSCs plus Tr1 cells showed slightly reduced IL-6 levels (not statistically significant) with a significant increase in TGF-β levels (Control vs. MSC+Tr1) in serum on day 7 (four weeks) following injection of MSCs with or without Tr1 cells in the CIA model (Fig. 2F).

### Reciprocal regulation of CII-specific Treg and Th17 cells by MSCs and Tr1 cells in CIA mice

**Figure 3:**
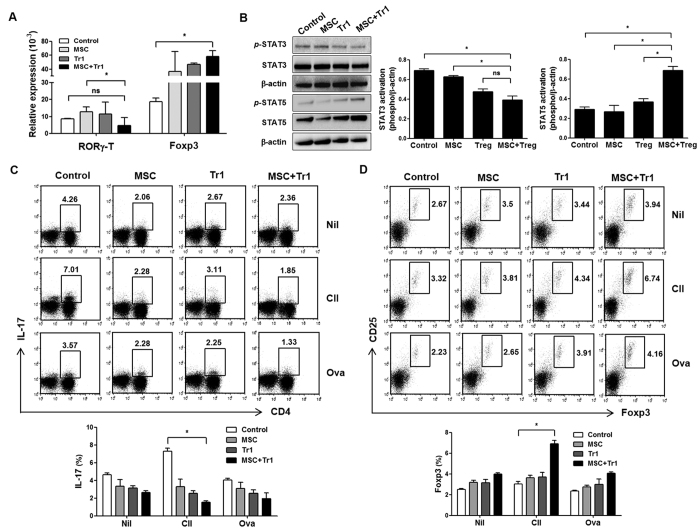
CII-specific Th17/Treg cell regulation by co-infusion of MSCs and Tr1 cells into CIA mice. (**A**) Four weeks following the primary immunization, mRNA expression levels of RORγ-T (n = 4) and Foxp3 (n = 4) were measured in splenic cells by real-time PCR (target/β-actin). (**B**) Splenic cells were isolated from each group and subjected to western blot analysis to determine levels of *p*-STAT3, *p*-STAT-5, STAT3, and STAT-5. (**C,D**) Draining lymph node cells from mice obtained from each of the groups were incubated with Nil, CII (40 μg/ml) or OVA (40 μg/ml)-stimulated cells for three days at the same time points. OVA was used as a negative control. The figure depicts intracellular staining for Foxp3 expression and IL-17 production and is representative of three independent experiments. Frequencies of CD4+IL-17+ T cells and CD25+Foxp3+ cells in the gated CD4+ T cell populations was assessed using intracellular flow cytometry. All graphs display the means ± SEM; results are representative of two independent experiments and statistical significance was determined by Student’s two-tailed *t*-test and ANOVA with Bonferroni correction for multiple comparisons (*p < 0.05).

Next, we examined whether co-infusion of MSCs and Tr1 cells contributes to Treg cells induction and inhibits the development of Th17 cells. Changes in Treg and Th17 cells related to transcription factors were investigated in splenic cells from CIA mice. RORγ-T plays an important regulatory role in thymopoiesis, by promoting thymocyte differentiation into pro-inflammatory Th17 cells^32^,^33^. Splenic cells from CIA mice exposed to MSCs plus Tr1 cells showed markedly increased expression of Foxp3 (Control vs. MSC+Tr1), but slightly decreased expression of RORγ-T (Fig. 3A; MSC vs MSC+Tr1).

To further increase our understanding of the signaling events involved in the reciprocal effects of co-infusion with MSCs and Tr1 cells in CIA mice, we analyzed the inhibition of STAT3, which limits Th17 differentiation, as well as the expression of STAT5, which induces Treg cells differentiation^34^. Two weeks following the second immunization, MSCs and Tr1 cell-treated CIA mice showed markedly increased expression of *p*-STAT5 compared with those treated with either MSCs or Tr1 cells alone, whereas *p*-STAT3 activity decreased compared with both the control and single cell therapy groups (Fig. 3B).

These data are consistent with our hypothesis that CD4+ T cells activation in the presence of CII stimulation induces CD4+CD25+Foxp3+ Treg cells differentiation, and that the upregulation of CD4+IL-17+Th17 cells is abolished after CII stimulation in the draining lymph node by co-infusion of MSCs and Tr1 cells to CIA mice compared with controls (Fig. 3C,D). Inhibition of IL-17 by co-infusion is associated with increased Foxp3 expression, suggesting that co-infusion reciprocally affects both Treg cells induction and Th17 cells differentiation. Thus, co-infusion of MSCs and Tr1 cells markedly alters the balance between Treg cells and Th17 cells in CIA mice, resulting in an altered disease course.

### Tr1 cells enhance TLR3 expression in MSCs

**Figure 4:**
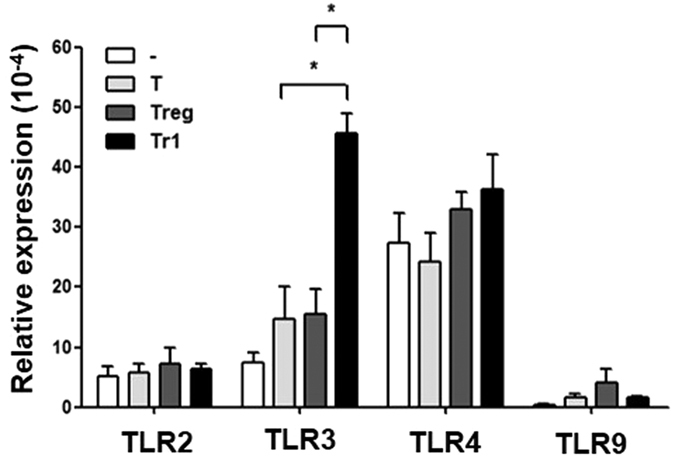
TLR mRNA expression by co-culturing MSCs with T, Treg, or Tr1 cells. TLR expression in MSCs was assessed using real-time PCR. MSCs were isolated by negative selection of CD4+ T cells in MSCs alone (n = 7) and co-cultured with T (anti-CD3, anti-CD28) (n = 4), Treg (anti-CD3, anti-CD28, anti-IFN-γ, anti-IL-4, recombinant TGF-β, and retinoic acid) (n = 7), or Tr1 (anti-CD3, anti-CD28, Dex, and Vit D3) (n = 7) cells co-cultured with MSCs for 24 h. All graphs display the means ± SEM; results are representative of two independent experiments and statistical significance was determined by Student’s two-tailed t-test and ANOVA with Bonferroni correction for multiple comparisons (*p < 0.05).

Previous studies have indicated a role for TLR activation in MSCs during the development of new therapeutic strategies for inflammatory or autoimmune diseases, because MSCs are likely activated through TLR ligands in sites of injury or inflammation^13^. Therefore, we hypothesized that TLR stimulation by Tr1 cells regulates MSC function. Changes in TLR expression in MSCs stimulated by T, Treg and Tr1 cells were investigated to explore the mechanisms involved in the Tr1-treated MSC-mediated immunosuppressive effects. To investigate the expression of TLRs in MSCs, which were sorted using flow cytometry from co-cultures of MSCs with T, Treg or Tr1 cells for 24 h, the expression of TLR2, TLR3, TLR4, and TLR9 were assessed using real-time PCR and flow cytometry. As a result, co-cultured Tr1 cells showed MSC-upregulated expression of TLR3 (T-treated MSC vs. Tr1-treated MSC, Treg-treated MSC vs. Tr1-treated MSC) (Fig. 4, Supplementary Fig. 2).

### MSCs upregulate TLR3 by IL-10-dependent expression of IFN-β in Tr1 cells

**Figure 5:**
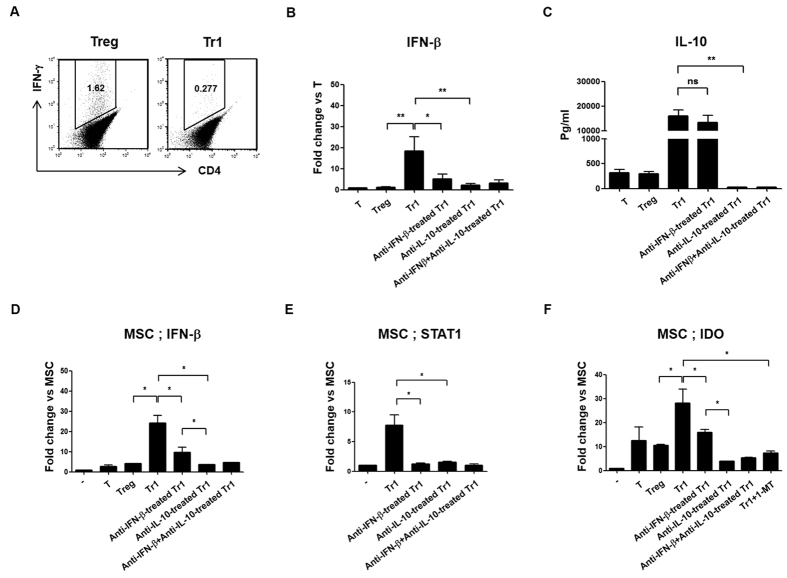
Production of IL-10-dependent IFN-β from Tr1 is required for IDO induction in MSCs. Induced T-cell stimulation method: T (anti-CD3, anti-CD28); Treg (anti-IFN-γ, anti-IL-4, TGF-β, retinoic acid, anti-CD3, anti-CD28); Tr1 (Dex, Vit D3, anti-CD3, anti-CD28); anti-IFN-β-treated Tr1 (anti-IFN-β, Dex, Vit D3, anti-CD3, anti-CD28); anti-IL-10-treated Tr1 (anti-IL-10, Dex, Vit D3, anti-CD3, anti-CD28); anti-IFN-β plus anti-IL-10-treated Tr1 (anti-IFN-β, anti-IL-10, Dex, Vit D3, anti-CD3, anti-CD28). *In vitro* differentiation of Treg and Tr1 cell: Treg CD4+ Foxp3^high^IL-10^low^ cells; Tr1 CD4+Foxp3^low^IL-10^high^ cells. (**A**) Treg and Tr1 cells was analyzed by flow cytometry for intracellular IFN-γ in gated CD4+ T cell populations after differentiation for two days. Values represent the percentage of positive cells. (**B**) Analysis of IFN-β mRNA expression was performed using real-time PCR, as described in ‘Methods.’ Fold change represents the expression ratio. (**C**) T, Treg, Tr1, anti-IL-IFN-β-, anti-IL-10- or anti-IL-IFN-β plus anti-IL-10-treated Tr1 cells were analyzed by ELISA to examine IL-10 secretion. (**D–F**) Real-time PCR was performed to measure IFN-β, STAT1 and IDO mRNA expression. MSCs were isolated by negative selection of CD4+ T cells in T-, Treg-, Tr1-, anti-IFN-β-treated Tr1, anti-IL-10-treated Tr1, anti-IFN-β plus anti-IL-10-treated Tr1, or addition of 1-MT plus Tr1 cells co-cultured with MSCs for 24 h. Data are presented as fold changes, which were calculated as the value of each group divided by the value of MSCs (target/β-actin). All graphs display the means ± SEM; results are representative of two independent experiments and statistical significance was determined by Student’s two-tailed *t*-test and ANOVA with Bonferroni correction for multiple comparisons (*p < 0.05; **p < 0.01).

Previous studies reported that TLR3 mRNA levels are significantly increased following stimulation with IFN-γ and IFN-β, and to a lesser extent, IL-1β^35^. Therefore, we investigated the production of IL-10, as well as IFN-γ and IFN-β, in the presence of Dex and Vit D3 in Tr1 cells. Tr1 cells did not cause IFN-γ secretion (Fig. 5A)^36^. Interestingly, IFN-β expression was significantly higher in Tr1 cells than in Treg cells, according to real-time PCR and ELISA (Fig. 5B, Supplementary Fig. 3A). Utilizing anti-IFN-β– and anti-IL-10-targeting Tr1 cells, IFN-β expression significantly decreased in anti-IFN-β-treated Tr1 cells, as well as anti-IL-10-treated Tr1 cells compared to Tr1 cells. Therefore, IL-10-dependent IFN-β expression in Tr1 cells was higher than in Treg cells (Fig. 5B). On the other hand, anti-IFN-β-treated Tr1 cells showed no differences in IL-10 production compared to Tr1 cells (Fig. 5C). Therefore, IL-10 secretion is likely produced through a pathway independent of IFN-β in Tr1 cells.

### Autocrine IFN-β-activation of STAT1 enhanced the upregulation of IDO in Tr1-treated MSCs

Previously it was reported that TLR activation of MSCs was involved in the induction of functional IDO via IFN-β activating STAT1^37^. To investigate the expression of IFN-β, STAT1 and IDO in MSCs, cells were sorted using negative selection CD4+ T cells from co-cultures of MSCs with T, Treg, Tr1, anti-IFN-β-Tr1, anti-IL-10-Tr1, and anti-IFN-β plus anti-IL-10-treated Tr1 cells for 24 h, after which gene expression was analyzed using real-time PCR. Tr1-treated MSCs highly expressed IFN-β mRNA compared to Treg-treated MSCs. In addition, co-cultured anti-IFN-β-, as well as anti-IL-10-treated Tr1 cells with MSCs, exhibited lower expression of IFN-β compared to Tr1-treated MSCs. Furthermore, co-cultured anti-IL-10-treated Tr1 cells with MSCs showed decreased expression of IFN-β compared to co-cultured anti-IFN-β-treated Tr1 cells with MSCs (Fig. 5D, Supplementary Fig. 3B).

Signaling required for IDO induction is associated with IFN induction and STAT1-dependent signaling^38^. Next, we investigated whether Tr1 cells induce STAT1 expression in MSCs. We observed an increase in STAT1 mRNA and protein levels at 24 h after co-culturing Tr1 cells with MSCs compared to co-culturing anti-IFN-β-treated Tr1 cells and/or anti-IL-10-treated Tr1 cells with MSCs (Fig. 5E, Supplementary Fig. 3C). Although STAT3 is a key mediator of the IL-10 response^39^, Tr1 cells did not induce STAT3 expression in MSCs (data not shown).

In a similar manner, Tr1-treated MSCs demonstrated significantly increased expression of IDO compared to Treg-treated MSCs (Fig. 5F, Supplementary Fig. 3D). These results suggest that IFN-β production by Tr1 cells resulted in significant induction of IDO expression, which is dependent on IL-10 signaling.

Tryptophan-degrading enzyme indoleamine-2,3-dioxygenase (IDO) has been implicated in potent immunosuppressive mechanisms. In our experiment, to further investigate whether IDO-mediated immunosuppressive effects were involved, we used the IDO inhibitor 1-methyl-D-tryptophan (1-MT), which inhibits mRNA expression of IDO through p38 mitogen-activated protein kinase (MAPK) and c-Jun N-terminal kinase (JNK) signaling^40^. As a result, Tr1-treated MSCs showed significantly decreased expression of IDO by 1-MT, suggesting that IDO is involved in the interaction between MSCs and Tr1 cells.

### IFN-β and IL-10 are involved in Tr1-induced upregulation of IFN-β, STAT1 and IDO via different pathways in MSCs

**Figure 6:**
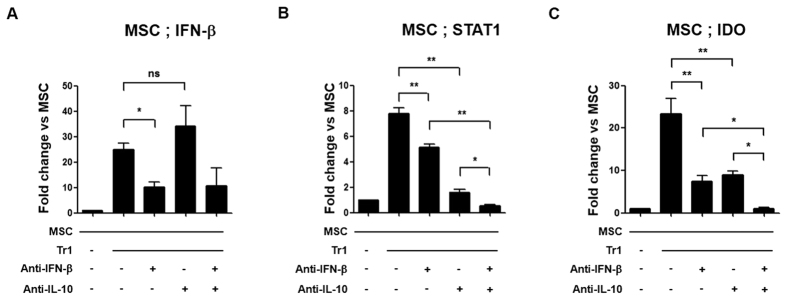
IL-10 and IFN-β synergistically induce IDO in MSCs via autocrine IFN-β and STAT1 signaling in Tr1 cells. (**A–C**) Real-time PCR was performed to measure IFN-β, STAT1 and IDO mRNA expression in MSCs using negative selection CD4+ T cells in co-cultured Tr1 cells and anti-IFN-β and/or anti-IL-10 pre-treated MSCs for 24 h (n = 6 per group). Data are presented as fold changes, which were calculated as the value of each group divided by the value of MSCs (target/β-actin). All graphs display the means ± SEM; results are representative of two independent experiments and statistical significance was determined by Student’s two-tailed *t*-test and ANOVA with Bonferroni correction for multiple comparisons (*p < 0.05; **p < 0.01).

To increase our understanding of the mechanisms associated with the immunuosuppressive effect of co-culture Tr1 cells with MSCs, we examined changes in IFN-β, STAT1 and IDO levels in Tr1-treated MSCs using IFN-β and IL-10-neutralizing antibodies. IFN-β and IL-10 share STAT1 as a signaling component. Therefore, we investigated whether these cytokines affect STAT1 activation and IFN-β production in MSCs. As a result, autocrine IFN-β production in Tr1-treated MSCs was inhibited compared to Tr1-treated MSCs, whereas IL-10 production did not affect autocrine IFN-β production in Tr1 cells (Fig. 6A).

In addition, STAT1 expression was decreased by use of anti-IFN-β and/or anti-IL-10 neutralizing antibodies in co-cultured Tr1-treated MSCs. When treated with both anti-IFN-β and anti-IL-10, a significant decrease in STAT1 expression was observed relative to incubation with anti-IFN-β or anti-IL-10 alone in co-cultured Tr1-treated MSCs (Fig. 6B, Supplementary Fig. 4A).

In a similar manner, anti-IFN-β and anti-IL-10 neutralizing antibodies significantly decreased IDO expression compared to treatment with a single neutralizing antibody in co-cultured Tr1-treated MSCs (Fig. 6C, Supplementary Fig. 4B). Therefore, these results suggest that IFN-β and IL-10 production by Tr1 affects the expression of IDO by activating STAT1 through different pathways in Tr1-treated MSCs.

## Discussion

The immunomodulatory properties of MSCs offer a potentially attractive therapeutic modality for autoimmune diseases^41^. Although MSCs are known to possess immunoregulatory functions, in a chronic inflammatory environment, MSCs may aggravate inflammation. Previous studies have suggested that IL-17 and TNF-α play a central role in RA, which results in reversion of MSC-induced immunosuppression, and the high levels of IL-6 in the presence of TNF-α may account for the lack of a beneficial effect of MSCs in CIA^17^,^42^,^43^. Therefore, the therapeutic functions of MSCs in CIA are insufficient to prevent overproduction of pro-inflammatory cytokines and partially reversed MSC-mediated immunosuppression of activated T cells proliferation.

To compensate for the disadvantages of MSC therapy, we previously reported a combination of MSCs and Treg cell therapy as a novel method for synergistic immunomodulatory effects on allograft rejection^44^, acute GVHD^45^ and induction of mixed chimerism^46^. Therefore, we speculated that a conditional microenvironment containing regulatory immune cells, such as Treg or Tr1 cells, might increase the immunoregulatory strength of MSCs in RA.

We first analyzed the effects of Treg and Tr1 cells as MSC partners in an experimental model of arthritis. As seen in Fig. 1A, the generation of Tr1 cells from naïve CD4+ T cells using both Dex and Vit D3 was first described by Barrat *et al.*^36^. These Tr1 cells stably produce IL-10, but show only marginal or no IL-2, IL-4 (data not shown) and IFN-γ production in stimulated and removed stimulated culture supernatants. Foxp3 is critical for both the development and function of Treg cells in mice (Fig. 1A). However, unlike CD4+ T cells from C57BL/6 mice, DBA1J-derived CD4+ T cells are resistant to TGF-β- and retinoic acid-induced Treg cells conversion^24^. It is possible that a small number of contaminating CD4+Foxp– T cells produce IL-17 in Treg cells after removing the stimulant (Fig. 1C). On the other hand, despite blocking IL-10 and IFN-β signaling, Tr1 cells did not produce IL-17 (data not shown), but stably produced IL-10 *in vitro* (Fig. 4C). Therefore, our study suggests that Treg cells may facilitate the conversion of CD4+Foxp3– T cells towards a CD4+IL-17+Th17 phenotype, whereas Tr1 cells do not induce the conversion of CD4+IL-10– Tr1 cells to CD4+IL-17+ T cells. Our results provide evidence that Tr1 cells are more stable and functional than Treg cells in mice with established autoimmunity.

The balance between Th17 and Th1 responses is important because any enhanced IL-17 activity could be secondary to robust Th-cell responses typical of RA. Moreover, Th1 cytokine IFN-γ is known to suppress Th17 development^47^. In addition, exposure to IFN-γ provides a ‘licensing’ step that is fundamental for the induction of MSC-mediated immunosuppression. Although Tr1 cells can generate IL-10 (but not IFN-γ) in the presence of Dex and Vit D3 when adoptively transferred in a CIA model, these cells prevent RA by slightly decreasing the expression of IL-17 (no statistical significance) via production of CD4+IFN-γ+ T cells compared to Treg-treated CIA mice (Fig. 1E). These data indicate that, because Tr1-treated CIA mice exhibit increased IFN-γ production *in vivo*, co-infusion of MSCs and Tr1 cells may induce an increase in IDO production in IFN-γ-licensed MSCs, and may therefore be applicable as a treatment for RA in a synergistic manner.

Previous reports suggest that MSCs inhibit Th17 cells, but they do not inhibit Treg cells; therefore, this effect must occur, at least in part, through IDO expression^29^. This study showed that the therapeutic effects of co-infusion of MSCs and Tr1 cells imply reciprocal regulation of CII-specific endogenous Treg and Th17 populations in draining lymph nodes and spleen cells (Fig. 3). Moreover, the secretion of TNF-α and IL-6 decreased while that of IFN-γ and TGF-β increased, in serum (Fig. 2F) and spleen cells (Fig. 2D), suggesting that co-administration of MSC- and Tr1 cell-mediated suppressive activity *in vivo* is partly attributable to soluble mediators. Furthermore, we believe that Treg-treated MSCs increase the production of TNF-α compared to Tr1-treated MSCs *in vitro* (data not shown), indicating that co-infusion of MSCs and Treg cells may reduce immune suppression during the development of RA compared to co-infusion of MSCs and Tr1 cells. For this reason, although there was no difference in CII-specific T cell proliferation between MSCs plus Treg (1: 1) or Tr1 (1: 1) cells, the suppressive effect was reduced from MSCs plus Treg (1: 2) compared to MSCs plus Tr1 (1: 2) cells and MSCs plus Treg (1: 1) cells following CII-specific stimulation (Fig. 2A). Although we did not assess the effect of co-infusion of MSCs plus Treg cells in an experimental mouse model of arthritis, co-administration of MSCs plus Treg cells is also expected to ameliorate arthritis, according to the results of CII-specific T-cell responses *in vitro*. However, we suggest that MSCs plus Tr1 cell therapy would be more effective in RA than MSCs plus Treg cell therapy.

Our results showed that the combination of MSCs and Tr1 cells induced a significant reduction in the incidence and severity of clinical arthritis, while MSCs suppressed the CII-specific T cell response *in vitro* but failed to prevent severe joint swelling and joint inflammation due to mononuclear cell infiltration *in vivo* (Fig. 2B–D). Although MSCs possess an immunosuppressive ability, this has recently been found to be not constitutive, and depends strongly on the prevailing inflammatory conditions. Thus, MSCs must be administered under conditions that maintain their immunosuppressive function.

Of note, our results suggested reciprocal regulation of CII-specific Treg and Th17 cells by MSCs and Tr1 cells in CIA mice (Fig. 3C,D). IL-6 contributes to the development of Th17 cells, but suppresses Treg cells differentiation in the presence of TGF-β. We hypothesize that co-infusion of MSCs plus Tr1 cells to CIA mice resulted in significantly increased production of TGF-β, but that of IL-6 decreased *in vivo*. However, serum TGF- β and IL-6 levels did not differ significantly between the MSC and MSC plus Tr1 groups (Fig. 2F). This is likely because MSCs exert an immunosuppressive effect by secreting TGF-β and IL-6^48^.

Previous studies have indicated the important role of TLRs in MSC immunomodulating properties, which may modulate MSC therapeutic potential *in vivo*^13^. Opitz *et al.* showed that MSCs pre-stimulated with TLR3 and TLR4 agonists showed enhanced IDO-mediated T cell suppression. The investigators defined a novel IFN-γ-independent pathway involving an autocrine IFN-β signaling loop that requires protein kinase R and STAT1/IRF1 activation^37^. In addition, according to Waterman *et al.*, TLR3 stimulation leads to the secretion of factors with mostly immune-suppressive properties, while stimulation of TLR4 with lipopolysaccharide (LPS) results in the secretion of more pro-inflammatory factors. Furthermore, when co-cultured with T cells, TLR4-primed MSCs augment T cell activation, whereas TLR3-primed MSCs suppress T cell activation^14^. We and other groups found that TLR3 is enhanced upon stimulation with IFN-β and/or IFN-γ^35^. Tr1 cells induced with Dex and Vit D3 express IFN-β (Fig. 5B), allowing for the induction of both endogenous (Fig. 5D) and exogenous IFN-β (Fig. 5B) in Tr1-treated MSCs. These results suggest that Tr1-treated MSCs induce TLR3 stimulation (Fig. 4), leading to alternate MSC2 polarization associated with anti-inflammatory resolution responses. According to this paradigm, MSC2 produces increased levels of IL-10, IDO and PGE_2_^14^. A comparative analysis of interactions between MSCs and Treg or Tr1 cells with a focus on PGE_2_, TGF-β and IL-10 was performed. However, there was no difference between Treg- and Tr1-treated MSCs in MSC-mediated immunoregulation (data not shown).

Tr1 cells primarily generate type I IFN-dependent IL-10^49^,^50^,^51^. However, as shown in Fig. 5C, IL-10 secretion is produced through a pathway independent of IFN-β in Tr1 cells. Tr1 cells may induce increased IDO expression by activating STAT1 signaling in a different pathway in Tr1-treated MSCs, according to studies using anti-IFN-β and anti-IL-10 antibodies. When treated with anti-IL-10, a greater decrease in STAT1 expression was observed relative to co-cultured Tr1-treated MSCs treated with anti-IFN-β alone (Fig. 6B). These results suggest that, since Tr1 cells produce higher levels of IL-10 compared to IFN-β, IL-10 inhibition strongly decreases STAT1 mRNA levels compared to inhibition of IFN-β (Fig. 6B). In addition, treatment of MSCs with anti-IFN-β-treated Tr1 cells significantly increased the clinical arthritis score and incidence of arthritis compared with MSC and Tr1 cell therapy (data not shown), suggesting that IFN-β plays an important role in IDO induction in MSCs.

**Figure 7:**
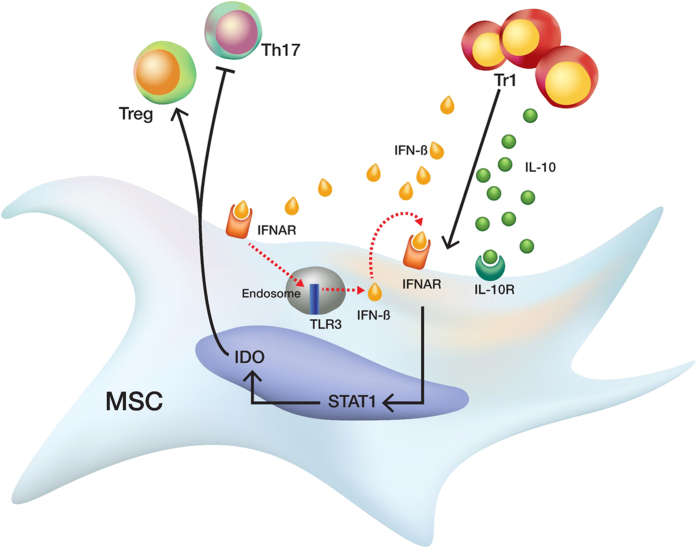
Schematic representation of the signaling pathway leading from Tr1 cell activation to the induction of IDO in MSCs. Tr1 cells activate STAT1 via IFN-β and IL-10, respectively. IFN-β induces the transcription of TLR3 which induces the secretion of IFN-β in an autocrine and paracrine manner. IFN-β may lead to transcriptional activation of IDO through the STAT1 signaling in MSCs; however, further studies are needed to confirm the dependency of IDO on STAT1 signaling. Production of IL-10 by Tr1 cells leads to activation of the STAT1 signaling. IDO activates Treg cells and suppresses Th17 cells.

In summary, we showed that co-infusion of MSCs and Tr1 cells exerts a synergistic immunoregulatory effect through increased IDO expression in MSCs via IFN-β and IL-10 production by Tr1 cells for the alleviation of RA development (Fig. 7). Thus, co-infusion of MSCs and Tr1 cells, which include highly active components of inflammation modulation and tolerance induction, may represent a new method for the treatment of RA in clinical trials.

## Methods

### Mice

Six-week-old male DBA/1 J mice (H-2q) were purchased from OrientBio (Seongnam, Korea) and maintained in the pathogen-free animal facilities of the Catholic University of Korea.

### Ethics

All procedures involving animals were in accordance with the Laboratory Animals Welfare Act, the Guide for the Care and Use of Laboratory Animals, and the Guidelines and Policies for Rodent Experimentation provided by the Institutional Animal Care and Use Committee (IACUC) of the school of medicine of the Catholic University of Korea. This study protocol was approved by the institutional review board of The Catholic University of Korea (CUMC-2012-0137-03).

### Isolation and culture of mouse bone marrow-derived MSCs

Six- to eight-week-old DBA1J mouse bone marrow cells were collected by flushing femurs and tibias with Dulbecco’s modified Eagle’s medium (Gibco, Carlsbad, CA, USA) containing 2 mM L-glutamine (Gibco), 1% antibiotics (penicillin (10 U/ml)–streptomycin (10 g/ml)) (Gibco) and 15% heat-inactivated fetal bovine serum (FBS, Gibco) with an endotoxin level ≤5 EU/ml and hemoglobin level ≤10 mg/dl (Gibco)^52^. When cells reached around 80% confluency, the medium was aspirated and 3–5 ml trypsin-EDTA (Gibco) were added to each dish. The dishes were then incubated for ~5 min to allow cell detachment. Next, an equal volume of culture medium was added to inactivate trypsin. The marrow cell immunophenotypes were persistently positive for Sca-1 (D7; BioLegend, San Diego, CA, USA), CD44 (IM7; eBioscience, San Diego, Ca, USA), and CD29 (HMβ1-1; BioLegend), but negative for c-Kit (2B8; BioLegend), CD11b (M1/70; BD Pharmingen, San Diego, CA, USA), CD34 (MEC14.7; BioLegend), CD106 (429 (MVCAM.A); BD Pharmingen), CD45 (30-F11; BD Pharmingen), CD31 (MEC 13.3; BD Pharmingen), CD80 (16-10A1, BD Pharmingen), and CD86 (2331 (FUN-1), BD Pharmingen) after more than 10 passages (two months of culturing) (Supplementary Fig. 1).

### Cell culture

To obtain Treg cells, CD4+ T cells were isolated from the spleens of control (untreated mice) or CIA mice in a DBA1J background, using microbeads conjugated to monoclonal anti-mouse CD4 antibodies (L3T4 MicroBeads; Miltenyi Biotech, Bergisch Gladbach, Germany). CD4+ T cells (5 × 10^5^) were cultured with plate-bound anti-CD3 (1 μg/ml; BD Pharmingen), soluble anti-CD28 (1 μg/ml; BioLegend), anti-IFN-γ (5g/ml; R&D Systems, Minneapolis, MN), anti-IL-4 (5 μg/ml; R&D Systems), human recombinant transforming growth factor beta (TGF-β) (5 ng/ml) (PeproTech, London, UK), and retinoic acid (0.1 μM) (Sigma-Aldrich, St. Louis, MO, USA) for two days^24^.

To obtain Tr1 cells, isolated CD4+ T cells (5 × 10^5^) from single-cell suspensions of splenocytes in control or CIA mice in a DBA1J background were cultured with plate-bound anti-CD3 (1 μg/ml), soluble anti-CD28 (1 μg/ml), vitamin D3 (Vit D3) 10^−7^ M (Sigma-Aldrich), and dexamethasone (Dex) 5 × 10^−8^ M (Sigma-Aldrich) for two days^36^.

MSCs (5 × 10^5^) were co-cultured with irradiated (2000 rad) T (5 × 10^5^), Treg (5 × 10^5^), or Tr1 (5 × 10^5^) cells (1:1 ratio) in 10 ml of culture medium for 24 h. MSCs were purified from co-cultures by sorting using negative selection of CD4+ T cells by flow cytometry.

### Induction and treatment of CIA

CIA was induced as described previously^53^. Briefly, DBA1J mice were injected in the tail base with 100 μg bovine type II collagen (CII, Chondrex, Redmond, WA, USA) emulsified in complete Freund’s adjuvant (CFA). Two weeks later, 100 μg CII with incomplete Freund’s adjuvant (IFA) was injected into the tail as a booster. Mice were injected intraperitoneally (i.p.) with 5 × 10^5^ MSCs and/or intravenously (i.v.) with 5 × 10^5^ Tr1 cells or intravenously (i.v.) with 5 × 10^5^ Treg cells 3 and 3.5 weeks (21 and 24 days) after the primary immunization. Control mice received i.p. or i.v. injections of an equal volume of phosphate-buffered saline (PBS, Gibco) at the same time points. The onset and severity of arthritis were determined by three independent observers, based on a scoring system described previously^54^. Twice weekly, the mice were examined visually for joint arthritis, and two independent observers who were blind to the experimental and control groups scored disease severity in each joint according to the following scale: 0, no evidence of erythema or swelling; 1, erythema and mild swelling confined to the mid-foot (tarsals) or ankle joint; 2, erythema and mild swelling extending from the ankle to the mid-foot; 3, erythema and moderate swelling extending from the ankle to the metatarsal joints; and 4, erythema and severe swelling encompassing the ankle, foot, and digits. The maximum possible score per mouse was 16 (4 joints × 4).

### Histological evaluation of CIA

Histological analysis was performed to determine the extent of joint damage. Mouse joint tissues were fixed in 4% paraformaldehyde, decalcified in EDTA bone decalcifier, and paraffin-embedded. Sections were then dewaxed using xylene and dehydrated through an alcohol gradient. Endogenous peroxidase activity was quenched with methanol/3% H_2_O_2_. Sections were routinely stained with hematoxylin and eosin (H&E) and evaluated blindly, as described previously^55^.

### Proliferation assay

CD4+ T cells (1 × 10^5^/well) from spleens of CIA mice were plated in 96-well-flat-bottomed plates (200 μl/well). Cells were then stimulated with anti-CD3 (1 μg/ml), anti-CD28 (1 μg/ml) and/or 1 × 10^4^ to 2 × 10^4^ MSCs and/or 1 × 10^4^ to 2 × 10^4^ Tregs, and/or 1 × 10^4^ to 2 × 10^4^ Tr1 cells for 72 h, followed by the incorporation of 1 μCi/ml [3H]-thymidine (GE Healthcare, Piscataway, NJ) for the last 18 h of the indicated culture period. Radioactivity was measured using a Micro Beta instrument (Pharmacia Biotech, Piscataway, NJ).

### Flow cytometric analysis

Single cell suspensions of spleen or draining lymph node were immunostained using various combinations of the following fluorescence-conjugated antibodies: CD4 (RM4-5; eBioscience), CD25 (37.51; BioLegend), Foxp3 (FJK-16 s; eBioscience), IL-17 (eBio17B7, eBioscience), TNF-α (MP6-XT22; BD Pharmingen), IFN-γ (XMG1.2; eBioscience), IL-4 (11B11; BD Pharmingen), and IL-10 (JES5-16E3; eBioscience). These cells were also intracellularly stained with the following antibodies: TNF-α, IL-17, IFN-γ, IL-10, and Foxp3. Prior to intracellular staining, cells were restimulated for 4 h with 25 ng/ml phorbol myristate acetate (PMA) (Sigma-Aldrich) and 250 ng/ml ionomycin (Sigma-Aldrich) in the presence of GolgiSTOP (BD Pharmingen). Intracellular staining was conducted using an intracellular staining kit (eBioscience) according to the manufacturer’s protocol. Flow cytometric analysis was performed on a FACS_LSR Fortessa (BD Pharmingen).

### Antigen-specific T cell stimulation

Draining lymph node cells (5 × 10^5^) from mice were incubated with Nil (unstimulated cell), CII (40 μg/ml) or ovalbumin (OVA, Sigma-Aldrich) (40 μg/ml)-stimulated cells for three days at the same time points. OVA was used as the negative control. The figure depicts intracellular staining for Foxp3 expression and IL-17 production and is representative of three independent experiments. Frequencies of CD4+IL-17+ T cells and CD25+Foxp3+ cells in the gated CD4+ T cells populations were assessed using intracellular flow cytometry.

### Enzyme-linked immunosorbent assay (ELISA)

Concentrations of IL-10, TGF-β, IL-6, and IL-17 were measured using a sandwich ELISA as follows. Anti-mouse IL-10, TGF-β, IL-6, or IL-17 monoclonal antibodies (R&D Systems) were added to a 96-well plate (Nunc, Roskilde, Denmark) and incubated overnight at 4 °C. Wells were blocked with blocking solution (PBS containing 1% bovine serum albumin and 0.05% Tween 20) for 2 h at room temperature. Test samples and standard recombinant mouse IL-10, IL-6, IL-17 and, human TGF-β (R&D Systems) were added to separate wells and the plate was incubated at room temperature for 2 h, after which the plate was washed. Biotinylated IL-10, TGF-β, IL-6, and IL-17 polyclonal antibodies (R&D Systems) were added, and the reaction was allowed to proceed for 2 h at room temperature. The plate was washed, ExtrAvidin–alkaline phosphate (1:2,000 dilution) (Sigma-Aldrich) was added, and the reaction was allowed to proceed for an additional 2 h. The plate was washed and 50 μl *p*-nitrophenyl phosphate disodium salt (Pierce Chemical Company, Rockford, IL), diluted to 1 mg/ml in diethanolamine buffer, and was applied. Experiments were performed according to the manufacturer’s instructions.

### Western blot analysis

Spleen cells were collected from DBA1J mice one week following cell therapy. Spleen cells were prepared from 5 × 10^6^ cells by homogenization in lysis buffer with a protease/phosphatase inhibitor cocktail (Cell Signaling, Danvers, MA) and centrifuged for 15 min at 14,000 revolutions per min. The protein concentration in the supernatant was measured by the Bradford method (Bio-Rad). Protein samples were separated by sodium dodecyl sulfate (SDS) gel electrophoresis and transferred to a nitrocellulose membrane (Amersham Pharmacia Biotech, Buckinghamshire, UK). Membranes were stained with primary antibodies specific to *p*-STAT3 (727), STAT3, *p*-STAT5, STAT5, or β-actin (Cell Signaling, Danvers, MA). HRP-conjugated secondary antibodies were then added. After washing with Tris-buffered saline and Tween 20 (TBST), hybridized bands were detected using an enhanced chemiluminescence (ECL) detection kit and Hyperfilm-ECL reagents (Amersham Pharmacia Biotech).

### Real time RT-PCR

Total RNA was extracted using TRIzol-LS reagent (Invitrogen). Total RNA (2 *μ*g) was reverse transcribed at 50 °C for 2 min, followed by 60 °C for 30 min. Quantitative PCR was performed using the FastStart DNA Master SYBR Green I kit and a LightCycler 480 Detection system (both from Bio-Rad, CA, USA), as specified by the manufacturer. The crossing point (Cp) was defined as the maximum of the second derivative from the fluorescence curve. Negative controls were included and contained all elements of the reaction mixture, except for template DNA. For quantification, relative mRNA expression levels of specific genes obtained using the 2^−ΔCt^ method are reported. β-actin, a housekeeping gene, was used for normalization. The following gene-specific primers were used: IFN-β (forward: 5′-AGCTCCAAGAAAGGACGAACAT-3′; reverse: 5′-GCCCTGTAGGTGAGGTTGATCT-3′), RORγ-T (forward: 5′- TGTCCTGGGCTACCCTACTG-3′; reverse: 5′-GTGCAGGAGTAGGCCACATT-3′), Foxp3 (forward: 5′-GGCCCTTCTCCAGGACAGA-3′; reverse: 5′-GCTGATCATGGCTGGGTTGT-3′), IDO (forward: 5′-ACACATACGCCATGGTGATG -3′; reverse: 5′-CGGACTGAGAGGACACAGGT-3′), STAT1 (forward: 5′- AAGCGAACTGGATACATCA-3′; reverse: 5′-AAGCGAACTGGATACATCA-3′), TLR2 (forward: 5′-TGCTTTCCTGCTGGAGATTT-3′; reverse: 5′-TGTAACGCAACAGCTTCAGG-3′), TLR3 (forward: 5′-TTGTCTTCTGCACGAACCTG-3′; reverse: 5′-CGCAACGCAAGGATTTTATT-3′), TLR4 (forward: 5′-CAAGAACATAGATCTGAGCTTCAACCC-3′; reverse: 5′-GCTGTCCAATAGGGAAGCTTTCTAGAG-3′), TLR9 (forward: 5′-ACTGAGCACCCCTGCTTCTA-3′; reverse: 5′-AGATTAGTCAGCGGCAGGAA-3′) and β-actin (forward: 5′-GAAATCGTGCGTGACATCAA-3′; reverse: 5′-TGTAGTTTCATGGATGCCAC-3′).

### Statistical analysis

Statistical significance was determined using Student’s two-tailed *t*-test and one-way analysis of variance (ANOVA) with Bonferroni correction for multiple comparisons. Differences between arthritis incidences at a given time point were evaluated by χ^2^ contingency analysis. In all analyses, *P* values less than 0.05 were considered to indicate statistical significance.

## Additional Information

**How to cite this article**: Lim, J.-Y. *et al.* Enhanced immunoregulation of mesenchymal stem cells by IL-10-producing type 1 regulatory T cells in collagen-induced arthritis. *Sci. Rep.* **6**, 26851; doi: 10.1038/srep26851 (2016).

## Supplementary Material

Supplementary Information
