# Supplementary material for: Enhanced immunoregulation of mesenchymal stem cells by IL-10-producing type 1 regulatory T cells in collagen-induced arthritis
Source: Sci Rep. 2016 Jun 1;6:26851. doi: 10.1038/srep26851 (PMC4887998; doi:10.1038/srep26851)
Supplement: Supplementary Information [file srep26851-s1.doc]

**Enhanced immunoregulation of mesenchymal stem cells**

**by IL-10-producing type 1 regulatory T cells**

**in collagen-induced arthritis**

Jung-Yeon Lim,^1,2^ Keon-Il Im,^1,2^ Eun-Sol Lee,^1,2^ Nayoun Kim,^1,2^ Young-Sun Nam,^1,2^ Young-Woo Jeon,^1,2,3^ Seok-Goo Cho^1,2,3^

^1^Institute for Translational Research and Molecular Imaging; ^2^Laboratory of Immune Regulation, Convergent Research Consortium for Immunologic Disease; ^3^Department of Hematology, Catholic Blood and Marrow Transplantation Center, Seoul St. Mary’s Hospital, The Catholic University of Korea College of Medicine, Seoul, 137-701, Republic of Korea

**Supplemental Information-Lim et al.**

**Supplementary Methods:**

**Isolation and culture of mouse bone marrow-derived MSCs.** Six- to eight-week-old DBA1J mouse bone marrow cells were collected by flushing femurs and tibias with Dulbecco’s modified Eagle’s medium (Gibco, Carlsbad, CA, USA) containing 2 mM L-glutamine (Gibco), 1% antibiotics (penicillin (10 U/ml)-streptomycin (10 g/ml)) (Gibco) and 15% heat-inactivated fetal bovine serum (FBS) with an endotoxin level ≤ 5 EU/ml and hemoglobin level ≤ 10 mg/dl (Gibco)^1^. When cells reached ~80% confluency, the medium was aspirated and 3–5 ml trypsin-EDTA (Gibco) were added to each dish. The dishes were then incubated for ~5 min to allow cell detachment. An equal volume of culture medium was then added to inactivate trypsin. The marrow cell immunophenotypes were persistently positive for Sca-1 (D7; BioLegend, San Diego, CA, USA), CD44 (IM7; eBioscience, San Diego, Ca, USA), and CD29 (HMβ1-1; BioLegend), but negative for c-Kit (2B8; BioLegend), CD11b (M1/70; BD Pharmingen, San Diego, CA, USA), CD34 (MEC14.7; BioLegend), CD106 (429 (MVCAM.A); BD Pharmingen), CD45 (30-F11; BD Pharmingen), CD31 (MEC 13.3; BD Pharmingen), CD80 (16-10A1, BD Pharmingen), and CD86 (2331 (FUN-1), BD Pharmingen) after more than 10 passages (two months of culturing) (supplemental online Fig. 1). CIA mice received an intraperitoneal (i.p.) injection of 5 × 10^5^ MSCs/200 μl saline; the cells were placed on ice within 30 min after removal from culture.

**Mouse bone marrow-derived MSC differentiation to the adipogenic, osteogenic or chondrogenic lineage.** A sample of the MSCs was tested to confirm multipotency by culturing in various conditioned media that induced differentiation along either the adipogenic, osteogenic, or chondrogenic lineage using a mouse mesenchymal stem cell functional identification kit (SC010, R&D Systems, Minneapolis, MN).

**Flow cytometric analysis.** Single cell suspensions of MSCs were immunostained using various combinations of the following fluorescence-conjugated antibodies: CD4 (RM4-5; eBioscience) and TLR2 (6C2; eBioscience). These cells were also intracellularly stained with the following antibodies: TLR3 (11F8; BioLegend), TLR4 (UT41; eBioscience), TLR9 (M9.D6; eBioscience), IDO (mIDO-48; BioLegend), or Rat IgG2b (RTK4530; BioLegend). Intracellular staining was conducted using an intracellular staining kit (eBioscience) according to the manufacturer’s protocol. Flow cytometric analysis was performed on a FACS_LSR Fortessa (BD Pharmingen).

**Western blot analysis.** MSCs were prepared from 5 × 10^5^ cells in lysis buffer with a protease/phosphatase inhibitor cocktail (Cell Signaling, Danvers, MA) and centrifuged for 15 min at 14,000 revolutions per min. The protein concentration in the supernatant was measured by the Bradford method (Bio-Rad). Protein samples were separated by sodium dodecyl sulfate (SDS) gel electrophoresis and transferred to a nitrocellulose membrane (Amersham Pharmacia Biotech, Buckinghamshire, UK). Membranes were stained with primary antibodies specific to *p*-STAT1, STAT1, or β-actin (Cell Signaling, Danvers, MA). HRP-conjugated secondary antibodies were then added. After washing with Tris-buffered saline and Tween 20 (TBST), hybridized bands were detected using an enhanced chemiluminescence (ECL) detection kit and Hyperfilm-ECL reagents (Amersham Pharmacia Biotech).

**Enzyme-linked immunosorbent assay (ELISA).** Concentrations of IFN-β were measured using a sandwich ELISA as follows. Experiments were performed according to the manufacturer’s instructions (PBL Assay Science, Piscataway , NJ, USA).

**Supplementary Figure 1:**


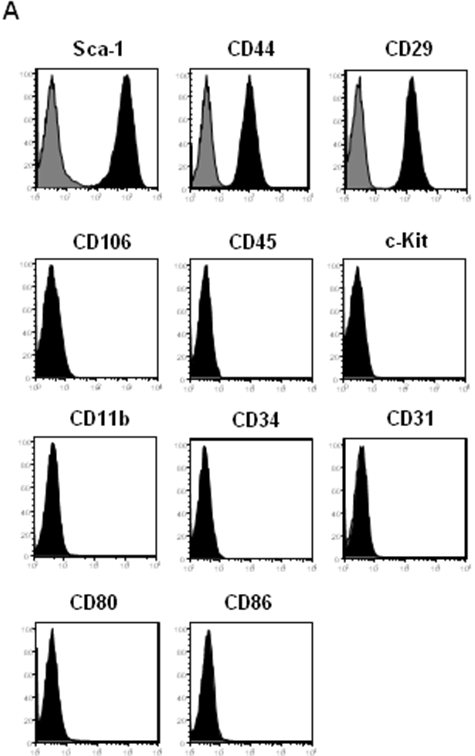

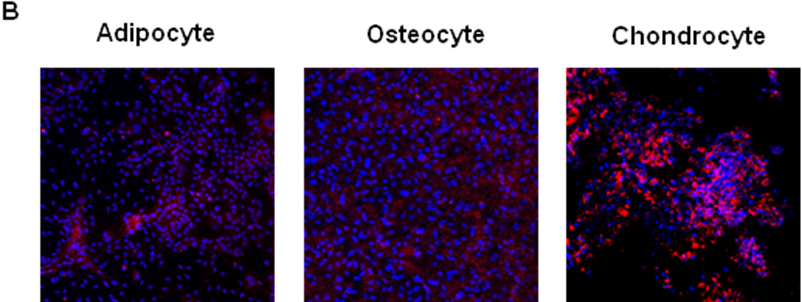


**Figure S1. Characterization and functional differentiation of mesenchymal stem cells (MSCs).** (A) MSCs were distinguished from hematopoietic cells by negative expression of the cell surface markers c-kit, CD11b, CD34, CD106, CD45, CD31, CD80, and CD86, but positive expression of Sca-1, CD44 and CD29. Results are representative of four independent experiments. (B) Staining of MSC cultures to demonstrate their adipogenic, osteogenic and chondrogenic differentiation potential.

**Supplementary Figure 2:**


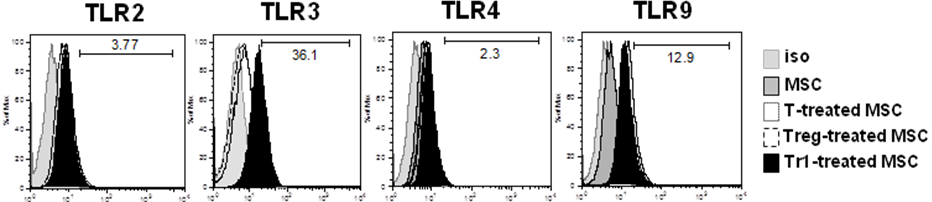


**Figure S2. Flow cytometry histograms showing expression of TLR2, TLR3, TLR4, and TLR9 expression in MSCs.** Isotype control is displayed as the grey line. Numbers indicate the percentage of cells in each gate for the TLR2, TLR3, TLR4, and TLR9 in Tr1-treated MSC group. Co-cultured Tr1 cells showed MSC-upregulated expression of TLR3 compared to T-, or Treg-treated MSC.

**Supplementary Figure 3:**

**
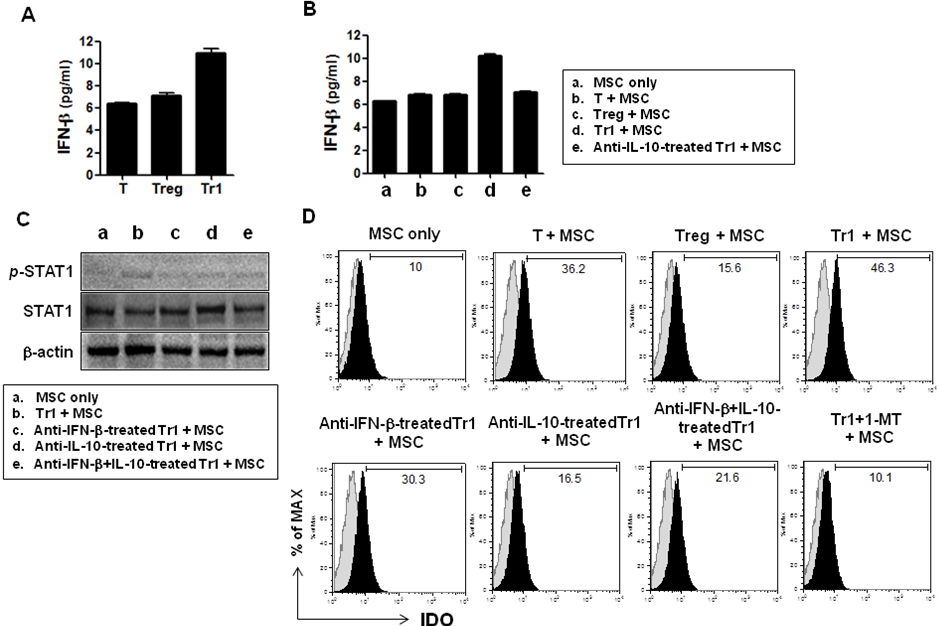
**

**Figure S3. Analysis of protein levels in cultured cells.** Induced T-cells stimulation method: T (anti-CD3, anti-CD28); Treg (anti-IFN-γ, anti-IL-4, TGF-β, retinoic acid, anti-CD3, anti-CD28); Tr1 (Dex, Vit D3, anti-CD3, anti-CD28); anti-IFN-β-treated Tr1 (anti-IFN-β, Dex, Vit D3, anti-CD3, anti-CD28); anti-IL-10-treated Tr1 (anti-IL-10, Dex, Vit D3, anti-CD3, anti-CD28); anti-IFN-β plus anti-IL-10-treated Tr1 (anti-IFN-β, anti-IL-10, Dex, Vit D3, anti-CD3, anti-CD28). (A) The supernatant levels of IFN-β in T, Treg, and Tr1 cells cultures after differentiation for two days were measured by ELISA. (B) The supernatant levels of IFN-β in T-, Treg-, Tr1-, or anti-IL-10-treated Tr1, co-cultured with MSCs for 24 h were measured by ELISA. (C) Western blot analysis was performed to measure *p*-STAT1, STAT1 and β-actin expression. MSCs were isolated by negative selection of CD4+ T cells in T-, Treg-, Tr1-, anti-IFN-β-treated Tr1, anti-IL-10-treated Tr1, or anti-IFN-β plus anti-IL-10-treated Tr1 co-cultured with MSCs for 24 h. (D) Flow cytometry was performed to analyze IDO expression in MSCs from T-, Treg-, Tr1-, anti-IFN-β-treated Tr1, anti-IL-10-treated Tr1, anti-IFN-β plus anti-IL-10-treated Tr1, or addition of 1-MT plus Tr1 cells co-cultured with MSCs. Isotype control is displayed as the grey line. All graphs display the means ± SEM.

**Supplementary Figure 4:**


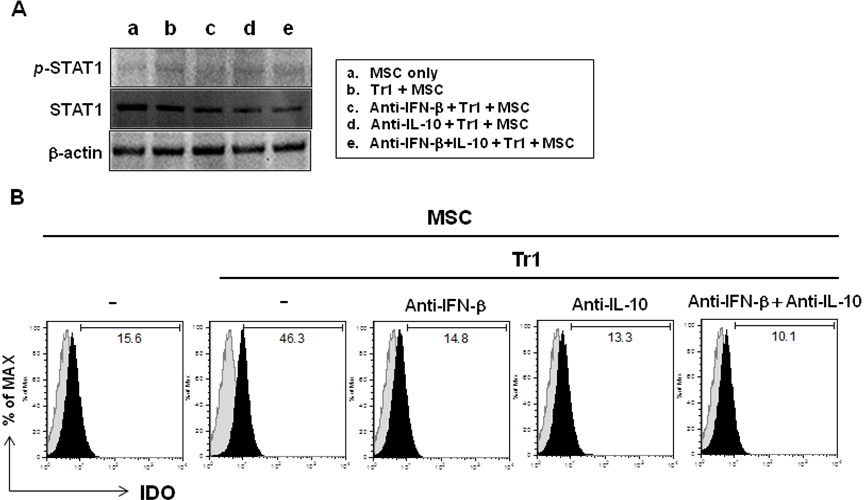


**Figure S4. Analysis of protein levels in MSCs.** (A) Western blot was performed to measure *p*-STAT1, STAT1, and β-actin expression in MSCs using negative selection CD4+ T cells in co-cultured Tr1 cells and anti-IFN-β and/or anti-IL-10 pre-treated MSCs for 24 h. (B) Flow cytometry was performed to analyze IDO expression in MSCs from co-cultured Tr1 cells and anti-IFN-β and/or anti-IL-10 pre-treated MSCs for 24 h. Isotype control is displayed as the grey line.

**Reference**

1. Soleimani, M. & Nadri, S. A protocol for isolation and culture of mesenchymal stem cells from mouse bone marrow. Nature protocols 4, 102-106, doi:10.1038/nprot.2008.221 (2009).
